# Antitoxin in Cerebro-Spinal Meningitis

**Published:** 1900-10

**Authors:** Francis M. O’Gorman

**Affiliations:** Buffalo, N. Y., The Carmichael


### ANTITOXIN IN CEREBRO-SPINAL MENINGITIS.

By FRANCIS M. O'GORMAN, M. D., Buffalo, N. Y.

I WAS summoned in great haste about eight o'clock one morning to see an infant who, the messenger said, was dying of convulsions. I hastened to the house and found a six months old child in a comatose condition, from which it would arouse with a general clonic convulsion, lasting from two to five minutes. Its temperature was 105°; respirations, 95, and pulse 190. The breathing was loud and stertorous, easily heard in any part of the house, all the accessory muscles of respiration being brought into action in its attempt to obtain air. Hardly any costal breathing existed, but the abdominal was greatly exaggerated. A sharp, ringing, brassy cough, followed by an attempt to inspire, invariably threw the child into a spasm. The family said that the child had had a severe cold for about a week. I immediately put the child in a warm bath and gave it fifteen grains of sodium bromide and a few drops of tincture of belladonna. These measures seemed to relieve her for a few moments, but she would be seized by another convulsion from an apparent effort to breathe. I decided that there was an obstruction in the larynx and introduced my finger well into the child's throat, and removed a great quantity of mucus and what looked like fibrous membrane. The result was that the breathing became easier. The convulsive seizures, however, came on in a short time, and I relieved the child again with my finger. I then gave it a teaspoonful of syrup of ipecac by mouth and half a drachm of sodium bromide per rectum, These drugs were also fruitless. Warm baths, mustard footbaths. ice cap and antispasmodics were ineffectual. I resorted to chloroform, and while it was producing its quieting influence, I examined the child's chest and found tubular breathing and large moist râles. I hunted in vain for an area of vesicular breathing. Percussion revealed a dulness all over. Taking into consideration the apparent obstruction in the throat, the physical examination, the pulse, temperature and respiration, I decided that the child had a broncho-pneumonia and laryngeal diphtheria.

I sent for 1,000 units of anti-diphtheritic serum, and in the meanwhile applied mustard poultices to the thorax, back and front. When the serum arrived, I injected it all at once. I watched the effect of the antitoxin carefully. In two hours the temperature had fallen to 103°; the respirations to 86; the convulsions had almost ceased, but there was no change in the pulse. About four o'clock in the afternoon she had her last convulsion. At six I injected 500 units of antitoxin and subsequently the breathing became easier. The child remained in the stupor all day and night. The next morning its breathing again became difficult, whereupon I injected another 1,000 units of antitoxin, with the same result as previously stated. During the day the nurse noticed some twitching of the limbs. Some difficulty in swallowing nourishment was evident. Along the latter part of the day I injected 500 units more of antitoxin, making in all

3,000 units administered in thirty-six hours. Its beneficial influence was manifested in the absence of the convulsions and twitchings, and the ease with which she breathed, although the rapidity was not much diminished. I then began to administer oxygen, attaching the tube to a rubber nipple, which the child was in the habit of holding in her mouth. The oxygen was continued uninterruptedly for four days and then for fifteen minutes during every hour. After the first week it was given every two hours and then three or four times daily. The breathing became easier and slower, but did not go below 72 for 10 days. When it did begin to come down, it did in jumps of 10 and 14, but it hovered around 40 for several days. At the end of the third week it was between 20 and 30, and the child seemed to be in good condition. Its temperature had fallen after the first week to about 100° and gradually came to normal. The pulse was the alarming factor. It remained between 150 and 182 for about two weeks, and then fell to 140, where it anchored, even after the lungs were cleared up. All during this illness the child took its nourishment and retained it. Its bowels and kidneys needed no stimulation. Recovery was complete. The child became as fat, healthy and good-natured as ever. The extraordinary rate of pulse and respiration were the puzzling points in this case; also the manner in which they fell to normal or thereabout. The child enjoyed the best health for two months. Then I was called at three o'clock in the morning and found the child in convulsions more violent than those of two months previous. I saw the child the day before and it appeared well. Urethane was given in one grain doses every hour which seemed to relieve her along in the afternoon, when a consultation was held and my diagnosis of cerebro-spinal meningitis confirmed. During the night urethane lost its power and chloroform was resorted to. It eased it somewhat, but not entirely; the patient died at four in the afternoon. The convulsions previous to death were terrific, rolling of the head and eyes, photophobia and opisthotonos, being very well marked. I believe that the extraordinary pulse and respiration of the previous illness was due to a latent inherited meningeal inflammation, because the father has since given me a history of the disease in his family. I am fully satisfied that the antitoxin had a beneficial effect upon the convulsions in the previous attack, and also that the child had diphtheria and pneumonia. Three physicians who saw the case corroborated my diagnosis. A question upon which I invite comment is: did the antitoxin administered in the first attack have any influence upon the pulse, respirations and convulsions, which I

believe to have been due to meningeal imflammation? also, am I right in thinking that had an antitoxin been used in the last attack the child might have recovered?

THE CARMICHAEL.

